# A Scoping Review of Theoretical Lenses and Methodological Approaches in Indigenous Women’s Health and Well-Being Research in North America over the Past Two Decades

**DOI:** 10.3390/ijerph20085479

**Published:** 2023-04-12

**Authors:** Tricia McGuire-Adams, Janice Cindy Gaudet, Keira A. Loukes, Celeste Ferreira

**Affiliations:** 1 Faculty of Education, University of Ottawa, Ottawa, ON K1N 6N5, Canada; 2 Campus Saint-Jean, University of Albert, Edmonton, AB T6G 2R3, Canada; 3 School of Outdoor Recreation, Parks & Tourism, Lakehead University, Thunder Bay, ON P7B 5E1, Canada; 4 School of Human Kinetics, University of Ottawa, Ottawa, ON K1N 6N5, Canada

**Keywords:** Indigenous women’s health and well-being, cultural resurgence, critical theory, Indigenous women’s wellness

## Abstract

Theoretical approaches influence research design, engagement, and outcomes. The relevance of critical theoretical and methodological approaches to Indigenous women’s health and well-being research has increased in the last decade. It is difficult to assess the ways in which theoretical lenses can effectively interrupt and challenge systemic erasure, ongoing harms, and deficit-based (ill-health-centered) approaches to Indigenous women’s health and well-being, a fact that is not broadly acknowledged. We conducted a scoping review to (a) map the type and frequency of critical theoretical lenses used by researchers focused on Indigenous women’s health and well-being in North America over the past two decades and (b) identify which topics tend to use which theoretical lens. We have conducted a scoping review to examine peer-reviewed articles from eight electronic databases. In the articles selected over 2000–2021, we found an increase in the use of community-based participatory research, decolonial lenses, and feminist lenses. Over the last decade, there has been a decrease in quantitative social science approaches. While a range of critical theoretical and methodological approaches are increasingly being applied, the use of cultural resurgence and Indigenous feminism in health research is not widespread.

## 1. Introduction: Current Research Approaches to Indigenous Women’s Health

The health and well-being of Indigenous women are critical to the health and well-being of generations of Indigenous lives and lands. We know that Indigenous women influence and inform identity through the connection to kin, human, and non-human relatives. Child [1] best describes this as “holding worlds together”. Stories of creation, language, knowledge keepers, living experiences, and Indigenous women’s political, social, and economic responsibilities deepen this knowledge. Increasingly, gender is being affirmed as a social determinant of Indigenous peoples’ health [2,3,4,5]. This is due to decades of Indigenous women’s research that centers on “restoring balance” as a guiding principle to community sovereignty, self-determination, resistance, and cultural intelligence [6,7]. Cultural resurgence frameworks resist “deficit analysis”, discrepancies, and the “Indigenous problem” [7] (p. 21). Life teachings, kinship systems, wisdom practices, deep attention, love, collaboration, and community care are foundational to resurgence. There is extensive research demonstrating the historical ways in which gender discrimination continues to distance Indigenous women from their kinship roles and responsibilities. Colonial logic, and its tactic of interrupting women’s kinship connections, interferes with balancing Indigenous health and well-being.

Our shared research interest in centering Indigenous women’s health and well-being has called forth a deepened critical examination of the deficit-based Indigenous health research [8]. By “deficit-based”, we are referring to a colonial ideology that informs research design, including research questions, methodologies, analysis of results, and findings, which continues to pathologize Indigenous women and position health issues as problems that need to be fixed with Western interventions [9]. McGuire-Adams [8] argues that deficit thinking fixates on various degrees of ill health without the deep consideration of embodied settler-colonialism as the enduring political and social system of ongoing Indigenous elimination. Deficit thinking attributes individuals, kinships, leadership, and nations as solely responsible for, and/or deserving of, their own ill health. As such, critical theoretical perspectives offer a renewed perspective disrupting deficit-based shortcomings. Positioning ourselves in the research offers a regenerative viewpoint acknowledging our responsibility to people and place. As feminist, cis-gendered Anishnaabe, Métis, German–Scottish–Anishinaabe, and Black-mixed scholars, we are personally invested in a decolonial praxis as it is tied to our health and well-being and that of our respective relatives.

A neoliberal approach to Indigenous health research, which blames ill health on individual choices and decisions, works to conceal and distract from systemic failings. The positioning of self-inflicted ill health absolves settler colonial responsibility and justifies the multiple forms of violence Indigenous women have faced and continue to face [8,10,11]. In addition, settler-state denial of relationality to people, place, and land is celebrated and held up as a victory, a successful conquest of uncivilized and unoccupied lands. The colonial ideology that positions Indigenous peoples as the problem contributes to the systemic oppression that justifies violent inaction and action. What Tuck and Yang [12] refer to as “settler moves to innocence” divest any responsibility from the embodied colonial logic that creates, benefits from, and perpetuates systems of oppression, ongoing harm, continued erasure, and the exploitation of Indigenous women’s lives, bodies, and lands. As such, Indigenous health and well-being are conceptualized as being stuck in a perpetual loop of perceived helplessness and powerlessness, which reinforces the belief in their need to be saved. These narratives are rarely interrupted by decolonial logic, ontology, theories, and praxis, leaving a predominantly deficit-based landscape of Indigenous women’s health and well-being. Without culturally resurgent theoretical lenses to restore balance, this research is grossly inadequate. Thus, we conducted a scoping review to better understand which theoretical lenses have been used historically and are currently being used in Indigenous women’s health and well-being research. 

## 2. Methods

Our scoping review inquired into theoretical and/or methodological frameworks applied in Indigenous women’s health and well-being research over the last two decades. This work aims to inform current theoretical approaches in Indigenous women’s health and well-being research. Our methodological approach [13] follows an iterative six-step process as laid out by Arksey and O’Malley [14]. We also followed the Preferred Reporting Items for Systematic Reviews and Meta-Analysis extension for Scoping Reviews (the PRISMA-ScR) as updated by Peters et al. [15]. The steps we took are summarized as follows:

1. Identified the research questions as a collective. Our scoping review asked the following questions: Which theoretical lenses are used by researchers examining Indigenous women’s health and well-being in North America? What are the topics of research?
2. Completed an abstract analysis of the relevant articles published in academic journals. As our research questions are directed at the theoretical and methodological approaches of researchers, we limited our search to peer-reviewed articles in academic journals. We also limited our search to articles written in English;
3. Selected studies based on abstract analysis are to be included in a full-text analysis;
4. Charted the data gathered from selected studies after reading the full text;
5. Collated, summarized, and reported results.

Many scoping reviews also conduct a sixth step, which involves consulting with a wider community of scholars in the field to identify strengths and gaps in the analysis. This consultation will inform the next stage of our work as part of the creation of an anti-oppressive research tool and/or kinship-theoretical framework. It is the purpose of this paper to focus on the findings of the scoping review.

Peer-reviewed academic articles from the following databases were identified: Academic Search Complete; Native Health Database; Web of Science; Google Scholar; Bibliography of Native North America; Sociological Abstracts; Gender Watch; and Indigenous Peoples of North America. We used the following search terms:

1. Indigenous OR Aboriginal* OR Native* OR “American Indian*” OR “First Nations” OR Inuit OR Metis
  AND
2. woman OR girl* OR female* OR gender*
  AND
3. health* OR “mental health” OR well-being OR “well-being” OR well-being OR wellness OR medic* OR “physical activity” OR nutrition OR nutrient* OR “quality of life” OR illness
  AND
4. “North America” OR Canada OR America OR “United States” OR “USA”

### 2.1. Eligibility Criteria

**Figure 1:**
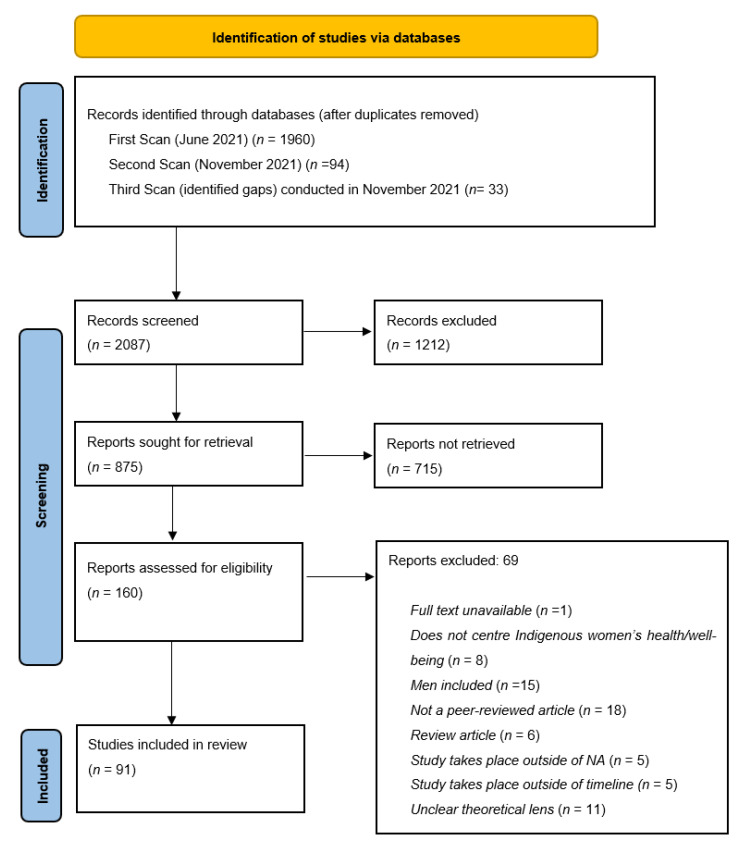
PRISMA flowchart detailing search retrieval and inclusion [16].

After collecting articles generated from these searches, two team members conducted two screenings by analyzing titles and abstracts of the gathered papers through a literature management tool, Zotero. The first screen’s eligibility criteria required that articles be published between January 2000 and June 2021, focused exclusively on Indigenous women based in North America and addressing questions related to health (physical, mental, spiritual, emotional) and/or wellness/well-being. The second screen was intended to reduce the articles to those published between 2011 and 2021 that explicitly stated their use of critical theories (e.g., Indigenous feminism, intersectionality, Indigenous resurgence, feminism, critical race) or community-based participatory research (CBPR). In this stage of our due diligence, the article list was reviewed by the two principal investigators, McGuire-Adams and Gaudet, to verify known scholarships that may have been missed as per our limitations, which we discuss in the forthcoming section. The two research assistants, Loukes and Ferreira, conducted a scan for eligible articles written by the scholars identified. In November 2021, we conducted a final search to capture any newly published articles. Figure 1 presents the number of articles initially captured and the screening process. 

### 2.2. Data Analysis and Synthesis

Coming to a consensus on inclusion or exclusion was based on a shared understanding of the criteria. Our final list of articles was categorized by theoretical and/or methodological approach, divided among the team members, and discussed collectively. We read the full text and extracted the following information from each article: author, title, abstract, journal, year, urban/rural, country, province/state, communities, nations, participants, Indigenous authorship, discipline, topic of study, research question, qualitative/quantitative/both, theoretical lens, methodology, methods, findings, and type of analysis. Our chart also included whether the research included intersecting forms of oppression, systemic racism, and colonization as a determinant of health and proposals, as well as our overall impressions. All of this data was organized using Google Sheets. The Results section captures theoretical lenses and methodologies that are being used in Indigenous women’s health and well-being research in North America. Articles using CBPR were included, given that its theoretical roots are informed by a critical lens and that it is so frequently applied in Indigenous health research.

## 3. Results

### 3.1. Search Yield

The initial search resulted in 2087 unique articles in peer-reviewed journals. Of these articles, 91 were included based on eligibility criteria. All were identified from academic databases, as indicated in Loukes et al. [13].

### 3.2. Theoretical Lenses and Methodological Approaches in Indigenous Women’s Health Research

**Figure 2:**
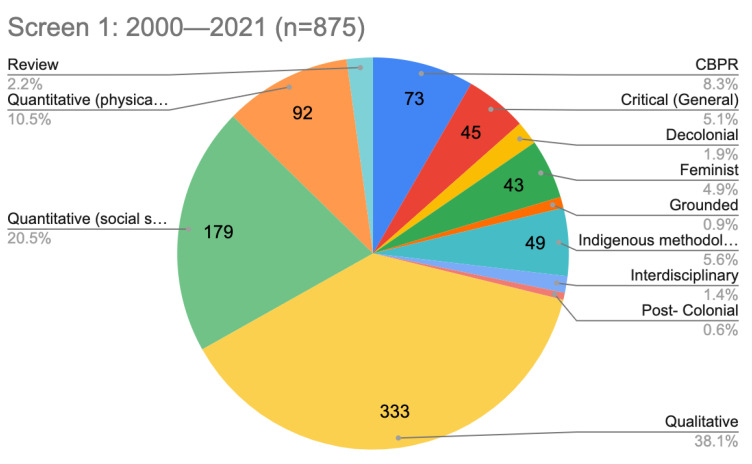
Breakdown of research approaches to Indigenous women’s health from 2000–2021.

**Figure 3:**
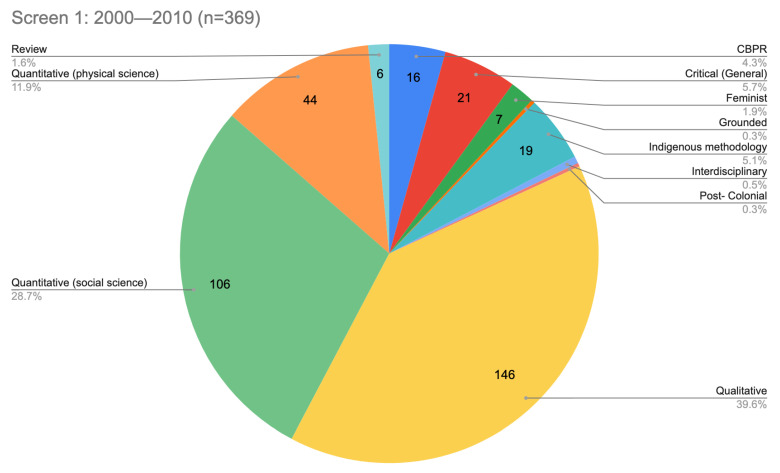
Breakdown of research approaches to Indigenous women’s health from 2000–2010.

**Figure 4:**
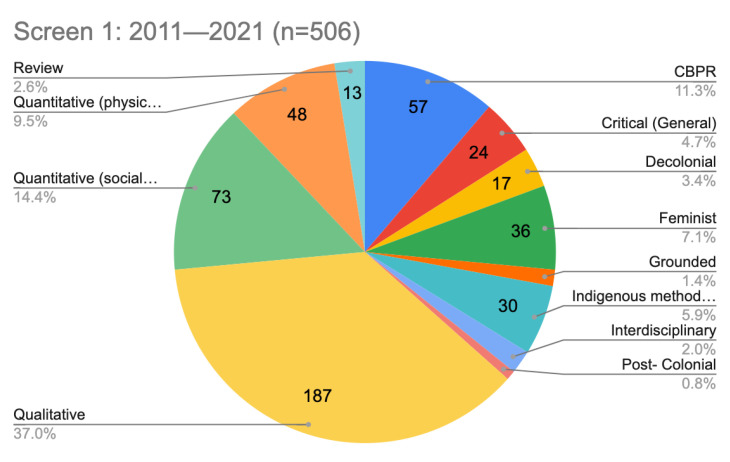
Breakdown of research approaches to Indigenous women’s health from 2011–2021.

**Figure 5:**
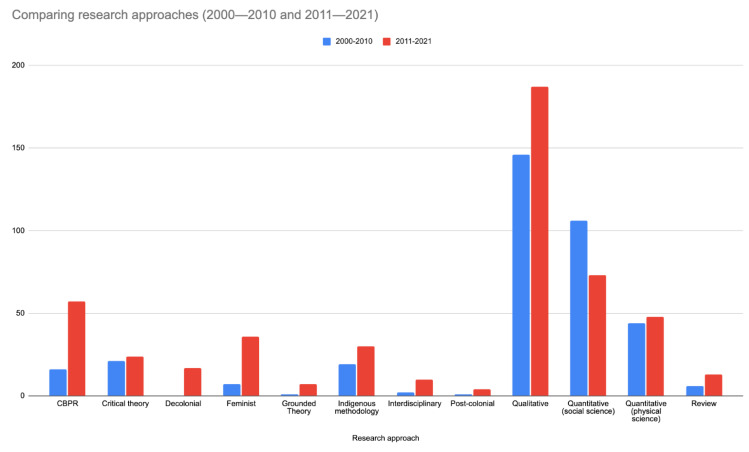
Comparison of theoretical lenses used in studying Indigenous women’s health in 2000–2010 and in 2011–2021.

Our analysis included a general overview of theoretical lenses and/or methodological approaches being used by researchers studying Indigenous women’s health from 2000–2021. The prevalence of various theoretical lenses used in Indigenous women’s health and well-being research from 2000–2021 is shown in absolute (Table 1), relative (Figure 2, Figure 3 and Figure 4), and comparative values (Figure 5). Figure 2, Figure 3 and Figure 4 show us that critical theories are still marginalized in Indigenous women’s health research. To capture a deeper understanding of how the uses changed over time, we separated data into two decades, 2000–2010 and 2011–2021. Figure 3 and Figure 4 show the relative proportion of each lens used in each decade, whereas Figure 5 shows an absolute comparison. 

These first results come from the abstract analysis. We collapsed theoretical lenses and methodological approaches under the following general headings. While CBPR and Indigenous methodologies are the two methodological approaches found in the abstract analysis, we decided to combine them with the theoretical lenses. We made this decision as both CBPR and Indigenous research methodologies, while methodologies are also used within critical theoretical lenses [17,18]. At this stage of the process, if the abstract contained more than one lens, we privileged the critical theory. For example, where theory was identified along with CBPR, the article was listed under its theoretical framework. See Appendix A for lists detailing collapsed categories per date range. 

**Figure 6:**
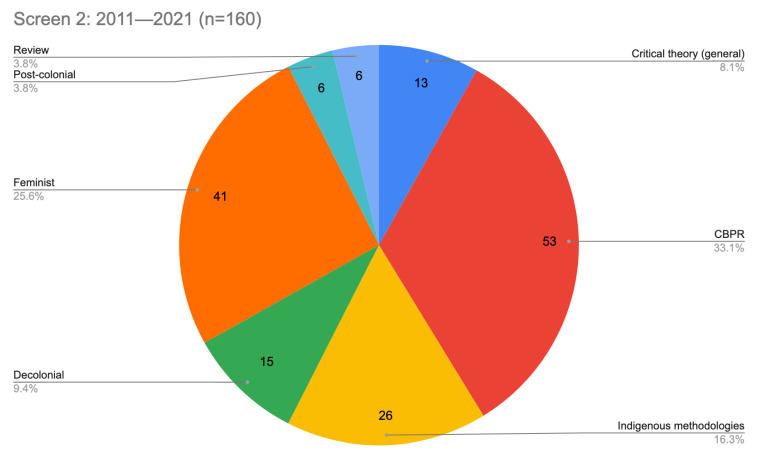
Categorizing critical research approaches to Indigenous women’s health from 2011–2021.

As this data set was extremely large, we made two decisions. The first was to focus on the last decade, 2011–2021. The second was to focus on Indigenous women’s health and well-being research that explicitly applied a critical theoretical approach or community-based methodological approach. Abstracts and titles were used to determine if the eligibility criteria were met. This screen resulted in 160 unique articles. The absolute and relative proportions of each critical lens used are shown in Table 2 and Figure 6, respectively. To simplify and streamline the result presentation, we grouped lenses under broad headings, as above. See Appendix A for a breakdown of which lenses are represented under each heading. 

Our final stage included a full-text read to explore the articles that met our inclusion criteria and to determine whether a critical discourse analysis would be beneficial for the next stage of our research. This full-text read identified further articles that fell under our exclusion criteria, as shown in Figure 1 (*n* = 160 to *n* = 91). We tracked various other elements, such as findings and self-location, which will serve for the critical discourse analysis study that the authors are currently conducting. In this phase, our results of the critical lenses used are displayed slightly differently, as we wanted to capture the frequency of each lens (Table 3). Some articles used multiple complementary lenses. 

Although there were only 91 articles, many studies used multiple lenses, which are all captured here, resulting in 134 data points.

We categorized the 91 articles that were included in the study by the lenses that authors explicitly stated were used. We found that in the earlier screens, using the abstracts and titles, categorizations were difficult as some articles used multiple lenses. We also needed to collapse some lenses under broader categories; for example, various forms of feminism were grouped under “Feminist”. We included all forms of feminism under this heading, including Indigenous feminism, as per bell hooks’ [19] concept that “feminism is for everybody”. This is not to erase the differences between feminists but to emphasize the similar overall approach to research that addresses white supremacist, capitalist, and hetero-patriarchal ideologies and their influences on collective health and well-being. 

### 3.3. Topics of Study

Another inquiry for our full-text analysis was the study topics of Indigenous women’s health and well-being research. The results are below in Table 4. 

While there tends to be a wide spread of topics, there is a greater proportion of studies on violence against Indigenous women and girls (particularly intimate partner violence) and maternal health (including pre-conception, prenatal, and breast-feeding). 

## 4. Limitations

Our research focused on critical Indigenous women’s health research. We relied heavily on authors explicitly stating their theoretical lens or methodology; therefore, we may have missed articles that used critical lenses without naming them as such. Another limitation is our focus on women exclusively, which in many ways works to uphold settler binaries of gender. While our search terms did include the term “gender” as a way to capture work with non-binary or transgender Indigenous folks, we did not explicitly include terms such as “Two-Spirit”, “transgender”, or “non-binary”, which may have resulted in missing a large body of decolonial and critical work. We recognize that health research that disrupts colonial and patriarchal views of gender is, too, part of reconstituting Indigenous women’s health and well-being. Likewise, articles that included Indigenous men’s health were not included, yet we understand that from an Indigenous kinship perspective, all of our relations are influenced by Indigenous women’s health. Finally, our searches were conducted entirely in English and may have missed key articles that were increasingly using Indigenous languages. 

### 4.1. Discussion

The summary of results (Table 1) revealed important trends in Indigenous women’s health research, including which theoretical lenses grew the most, which grew minimally, and which ones waned in use. The reader may also see Figure 2, Figure 3, Figure 4, Figure 5 and Figure 6 for visuals of these results. The results from the first screen revealed that from 2011–2021 compared to 2000–2010, there was a marked increase in the utilization of some theoretical lenses over others. CBPR, for instance, grew by 41, from 16 to 57 articles screened using CBPR. Similar growth was seen in the use of feminist theoretical frameworks; there were 7 studies using this approach in 2000–2010, and this grew to 36 in the 2011–2021 scan. Indigenous methodology and review frameworks grew from 19 to 30 and 6 to 13, respectively. Of note, decolonial approaches had zero uptake from 2000–2010, and from 2011–2021 this number grew to 17. Qualitative approaches grew from 146 in the first period to 187 in the second. Quantitative frameworks in physical sciences in the area of Indigenous women’s health grew only by 4 studies (44 to 48). Other frameworks that saw minimal growth during the two time periods were critical (general) lenses (21 to 24), grounded (1 to 7), interdisciplinary approaches (2 to 10), and post-colonial (1 to 4). The only decrease in the theoretical frame was the quantitative (Social Sciences) framework, which decreased from 106 in 2000–2011 to 73 in 2011–2021.

A key finding of these results, as demonstrated in Figure 2, is that among the theoretical frameworks used in Indigenous women’s health and well-being research spanning the entire screening period, 2000–2021, critical theoretical frameworks, such as critical (general), decolonial, grounded, feminist, Indigenous methodologies, post-colonial, and interdisciplinary, are marginalized. The more mainstream qualitative and quantitative (physical science and social science) frameworks comprise nearly two-thirds of the pie chart. The finding that quantitative research is one of the top three approaches to Indigenous women’s health research suggests that Western research approaches are still predominantly being used. These findings led the authors to question the broad rationale for the use of Western theories when studying the health of Indigenous women. The discursive political, economic, and socio-cultural contexts influence the choices for Western research approaches. There is an overreliance on Western approaches because, as McGibbon and Hallstrom [20] posit, there is a “political economy of health inequities” (p. 171) embedded in neoliberalist research and policies that re-inscribe colonial ideology, be it government, policy, research, or education. The favored theoretical frameworks and methodologies are those that continue the neoliberal colonial framework of settler colonial society. Notwithstanding the value in determining quantitative measures in Indigenous women’s health research, the absence of a critical lens from which to critique the neoliberal colonial ideology operationalized in research on Indigenous peoples, the reliance on Western research theories will remain unquestioned. This negation causes an overall lack of engagement with Indigenous resurgence practices such as the sovereignties of Indigenous knowledge, languages, and land-based healing [21]. This finding caused the authors to question whether it is time to consider the creation of an Indigenous women’s health and well-being discipline focused on the resurgence of education and research practices. We will consider this question more deeply in the next section.

The results also indicate that there is measured growth (from the first to the second period) in the amount and types of critical theoretical lenses being applied across Indigenous women’s health and well-being research articles. Of noted importance is the decrease in quantitative frameworks used in social science pertaining to Indigenous women’s health and well-being. This finding demonstrates that while the field of Indigenous women’s health and well-being research is indeed advancing the critical theoretical lenses necessary to better position the criticality of the field, overall, these lenses are still quite marginalized compared to the most popular lenses, general qualitative and quantitative approaches to research. A critical discourse analysis was not conducted at this time on the articles, and, therefore, we cannot determine to what extent, if any, the qualitative and quantitative studies took up any form of critical theory work in their studies.

**Figure 7:**
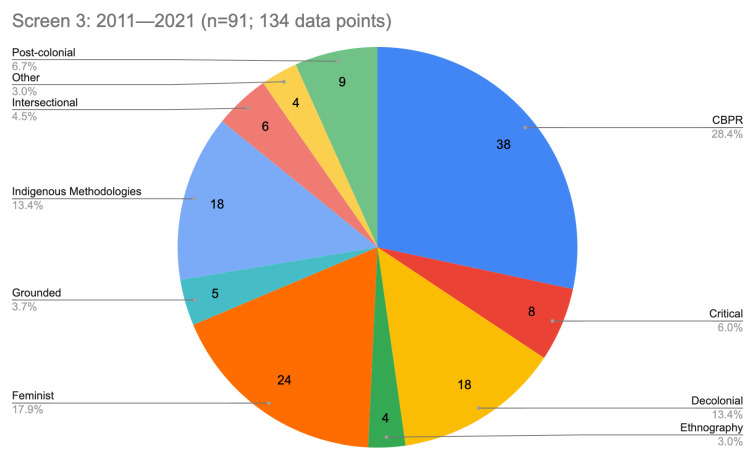
Proportion of included articles in each theoretical lens in Screen 3. Note that this reflects the frequency at that a lens was used. Sometimes, one paper used multiple lenses. In this analysis, each lens was counted.

Screen 3 focused solely on articles from 2011–2021 and included the critical theoretical frames noted in Screens 1 and 2, including critical theory (general), CBPR, Indigenous methodologies, decolonial, ethnography, feminist, post-colonial, and review articles (see Table 3 for frequency of use and Figure 7 for percentages). We found that CBPR is by far the most frequent critical lens, representing 28.4% of the total lenses captured. Feminist (17.9%) and Indigenous methodologies and Decolonial (13.4% each) were found to be the other highest lenses represented. While the rise of CBPR can be perceived as a movement toward more critical research, it could also be an indicator of an increase in in-name-only CBPR, where these methods have the potential to “re-inscribe power relations” [22] (p. 81), as for many, “participatory action research just represents the latest way to study [Indigenous people]” [23] (p. 140). Denzin et al. [24] point to the limitation whereby a critical theory fails to acknowledge Indigenous epistemologies as “sites of resistance and empowerment” (p. 9). Our next steps will work toward creating an anti-oppressive tool and/or kinship theoretical approach to Indigenous women’s health and well-being research. This will include a deeper text discourse analysis of the research articles (*n* = 91) to examine the ways Indigenous feminist theoretical approaches may disrupt the proliferation of Western lenses used in Indigenous women’s health research. The authors aim to enhance a research dialogue centering on the cultural resurgence and decolonial lenses to disrupt structures of settler colonialism and oppression and uphold resistance and agency as part of Indigenous women’s health and well-being.

Screen 3 also allowed us to reveal which topics were being covered in the use of critical theories. Refer to Table 4 to see the list of topics represented in the results. The highest-represented topics found in our scoping review include violence against women, maternal health, community-based perspective, Indigenous perspectives of health and well-being, cancer, and mental health. The highest-represented topics concern key health and well-being indicators. Violence against women and maternal health are key factors in addressing Indigenous women’s health and well-being overall. Our findings indicate that these topics are among the main foci of research on Indigenous women’s health and well-being. Resurgent critical research centering Indigenous women’s knowledge, body sovereignty, self-determination, Indigenous knowledge systems, diverse gender identities, and stories of resistance that aim to disrupt deficit-based research is less seen. Deficit-based research, as Hyett et al. [25] described, are those foci in research that “perpetuate deficit-based narratives” (p. 103). As such, of particular interest to the authors is the finding that many of the topics relate to studying and/or centering the health deficits of Indigenous women. 

### 4.2. Potential for the Creation of an Indigenous Women’s Health and Well-Being Discipline 

One of the goals of our full-text analysis was to determine which disciplines and/or fields of study were using critical theoretical lenses when studying Indigenous women’s health and well-being. We questioned the usefulness of this pursuit as placing articles in one category proved challenging as studies applying critical lenses to Indigenous women’s health tend to be interdisciplinary in nature, making visible the complexity and intersectionality of the inquiry. The research on Indigenous women’s health was not consistently distinct or easily attributed to one field of study. We discussed how we would determine the field, either by journal, author location, faculty, and/or research question. In some rare instances, the discipline was clear, while in the majority of others, it was very difficult to determine given the interdisciplinary nature of Indigenous women’s health research and would, thus, give an incomplete disciplinary picture of Indigenous women’s health. In these discussions, we questioned whether the results suggest that Indigenous women’s health and well-being can be, should be, or is a discipline on its own. Indeed, there remains an ongoing “need for more to be done in Indigenous health research and training” [21] (p. 186). We, thus, argue that the creation of an Indigenous women’s health and well-being discipline may be timely and needed. 

The results demonstrate that, while Indigenous health is ongoing across topics, there is an emergent potential for the development of discipline focused on Indigenous women’s health and well-being. This sentiment was also stated by Richmond [21] regarding the complexities involved in addressing Indigenous health inequities. She states, “The answers will not come merely from doing more (or spending more on) research but will be realized only through fundamental reorientation in *how we do* [emphasis in original] research and in creating the kinds of educational environments that can nurture these changes” [21] (p. 182). We argue that this emergent discipline could be rooted in Indigenous resurgence practices and values of rematriation, ceremony, land, language, kinship, and love. A disciplinary approach would train the next generation of Indigenous health researchers and practitioners to center Indigenous resurgence, rather than solely focus on Indigenous health deficits, in their practices of Indigenous health and well-being. Such a focused approach may amplify the critical theoretical frameworks currently marginalized in Indigenous health and well-being.

## 5. Conclusions

Our scoping review revealed that the use of critical and resurgent theoretical lenses is not widespread in Indigenous women’s health and well-being research. This could be due to the fact that of the articles we analyzed, Indigenous women’s health is uniquely positioned between the positivist and post-positivist approaches of the physical sciences, the quantitative approaches of social scientists, and the social justice approach of critical theorists. We recognize in our respective research that critical theory can be difficult to navigate as a decolonial and living practice. Some researchers may not have been exposed to critical theory and the ways in which theory is tied to resurgence, agency, and regenerative women’s health and well-being. What remains for the next stages of our analysis is to examine the ways in which some theoretical approaches center Indigenous women’s sovereignty, perspectives of health, and well-being and interrupt the camouflaging of compliance, protection of structures of power, and self-appointed allyship. In this context, this research team’s future goal is to create a theoretical tool and/or a kinship bundle to help guide researchers of all disciplines to conduct reflective, anti-oppressive, relational research that serves Indigenous women’s health and well-being in ways that are meaningful to Indigenous women.

## Figures and Tables

**Table 1:** The prevalence of theoretical approaches used in Indigenous women’s health and well-being research from 2000–2021.

Approach	2000–2010	2011–2021	2000–2021
CBPR	16	57	73
Critical (general)	21	24	45
Decolonial	0	17	17
Feminist	7	36	43
Grounded	1	7	8
Indigenous methodology	19	30	49
Interdisciplinary	2	10	12
Post-colonial	1	4	5
Qualitative	146	187	333
Quantitative (social science)	106	73	179
Quantitative (physical science)	44	48	92
Review	6	13	19
Total	369	506	875

**Table 2:** List of broad critical theories and the number of articles that used this lens.

Broad Critical Lens	Number of Articles Using This Lens
Critical theory (general)	13
CBPR	53
Indigenous methodologies	26
Decolonial	15
Feminist	41
Post-colonial	6
Review	6
Total	160

**Table 3:** Frequency of each critical lens was used in our third screen of eligible articles.

Lens Used	Frequency of Use
CBPR	38
Critical	8
Decolonial	18
Ethnography	4
Feminist	24
Grounded	5
Indigenous methodologies	18
Intersectional	6
Other	4
Post-colonial	9

**Table 4:** List of Topics and Frequencies.

Topic	Frequency	Topic	Frequency
Violence against women	15	Intersectionality of gender	1
Maternal health	11	Midwifery	1
Community based perspective	9	Obesity with 2-eyed seeing	1
Indigenous perspectives of health and well-being	8	Place-based	1
Cancer (breast, cervical, survivors, screening)	8	Sexual health	1
Mental health (homelessness, substance use)	5	Criminalization	1
Health equity gaps	4	Family suicide	1
Fetal Alcohol Spectrum Disorder	3	Healing family violence	1
HIV prevention and care	3	Water	1
Coercive sterilization	2	Healthy body perspectives	1
HPV Screening	2	Indigenous girlhood	1
Trauma impacts	2	Gender non-confirming youth	1
Human trafficking	2	Smoking	1
Life history/stories	2		
Menstrual health (menopause)	2		

## Data Availability

Not applicable.

## Appendix A

**Table A1:** Collapsed Categories for Screen 1, 2000–2010.

CBPR	Critical Theory	Decolonial	Feminist
PAR	Anti-oppressive	Decolonial	Eco-feminism
	Foucaultian	Decolonial action	Indigenous feminist
	Epistemic Injustice		Intersectionality
	Human rights based		
	Trauma-informed		

**Table A2:** Collapsed Categories for Screens 1 and 2, 2011–2021.

CBPR	Critical Theory	Decolonial	Feminist	Indigenous Methodology	Qualitative
PAR	Anti-oppressive	Decolonial	Feminist	Algonquin sweetgrass story weaving	Archival
	Epistemic Injustice	Decolonial action	Indigenous feminist	Cultural resurgence	Case study
	Human rights based		Intersectionality	Two-eyed seeing	Interpretive phenomenological analysis
	Trauma-informed				Historical analysis
					Democratic deliberation
					Governance transformational
					Holistic/inclusive
					Narrative
					Synthesis
					Self-determination
					Socio-ecological

**Table A3:** Collapsed Categories for Screen 3—Critical Theories 2011–2021.

Critical Theory	Indigenous Methodologies	Feminist
Anti-oppressive	Cultural resurgence	Feminist
Place-based methodologies	Tribal-critical race	Indigenous feminist
Trauma-informed		Intersectionality

**Table A4:** Collapsed Categories for Screen 4—Full-Text Analysis of Critical Theories.

CBPR	Indigenous Methodologies	Feminist	Decolonial	Critical Theory	Other
PAR	Metis Life Promotion	Decolonial feminist	Critical decolonizing lens	Critical victimology	Photovoice
CBPR	Indigenous research methodologies	Indigenous Feminist	Decolonizing praxis	Theory of ethical space	
Community-engaged	Anishinaabeg intelligence and Anishnaabee language. Anishaabeg research paradigm	Intersectionality		Alienation theoretical framework	
Community of practice (CoP) approach	Anishinaabeg research methodology applied principles of Anishinaabe-bimaadiziwin	Critical feminist analysis		Conceptual framework: The indigenist stress- coping model	
	Indigenous feminist	Gender analysis		Discourse analysis	
		Critical feminist theoretical perspectives			
